# Supplementary material for: Molecular cause and functional impact of altered synaptic lipid signaling due to a prg‐1 gene SNP
Source: EMBO Mol Med. 2015 Dec 15;8(1):25–38. doi: 10.15252/emmm.201505677 (PMC4718157; doi:10.15252/emmm.201505677)
Supplement: Supplementary file 1 — Appendix [file EMMM-8-25-s001.pdf]

Appendix

Table of contents

Appendix table S1 Basic socio-demographic variables

Appendix Supplementary Methods

Electrophysiological measurements

Surgical preparation and in vivo recordings

Immunocytochemistry

Mass Spectrometry analysis of C17 LPA incorporation and degradation

Mass Spectrometry assessment of LPA-species in the CSF.

Quantitative phosphorylation assessment

O-glycosylation assessment

TF-LPA uptake experiments

Behavioral analyses

Appendix References

Appendix table S1.

Basic socio-demographic variables

	prg-1 wt/wt; N=1799	R345T carriers; N=12	P value
Number of females (%)	1027 (57.1)	7 (58.3)	0.931*
	Mean (Std)	Mean (Std)	
Age (years)	36.0 (13.5)	28.4 (8.6)	0.071#
Cigarettes per day	6.2 (10.0)	4.3 (7.0)	0.718#
Years of school education	11.8 (1.5)	12.1 (1.3)	0.372#
Verbal IQ	109.8 (13.3)§	110.8 (12.9)	0.707#

wt=wildtype; *Chi-square test; #Mann-Whitney U test; §only available for 89.4% of wt/wt-controls.

Appendix Supplementary Methods

Electrophysiological measurements

Slice preparation and in vitro electrophysiology

Thalamocortical slices (350 µm) from in utero electroporated mice were prepared from 16-19 day old mice as described (Agmon & Connors, 1991). *In utero* electroporated neurons were identified by GFP-fluorescence. Hippocampal slices (250 µm) from PRG-1$^{+/-}$ mice were prepared from 16-19 day old mice.

Recordings were performed at 32-34°C (thalamocortical slices) or at room temperature (hippocampal slices) using borosilicate glass electrodes filled with (in mM): 110 potassium gluconate, 20 KCl, 5 NaCl, 0.5 $CaCl_2$, 5 EGTA, 20 HEPES, 2 MgATP, 0.3 GTP. pH was set to 7.2 with KOH. Electrophysiological signals were filtered at 3 kHz and sampled at a rate of 10 kHz. The holding potential was set to -70 mV. mEPSCs were recorded in the presence of TTX (1 µM; Alomone Labs, Jerusalem, Israel) and gabazine (10 µM, Sigma-Aldrich, Germany). eEPSCs were elicited at a frequency of 0.1 Hz (using an electrode placed in the thalamic VPM) and a pulse duration of 0.2 ms. QX 314 (2 mM) was added to the intracellular solution to prevent generation of action potentials in the tested neurons. Minimal stimulation

was accepted if eEPSCs occurred at short and constant latency (<3.5 ms), demonstrated paired pulse depression, and showed no decrease in latency of the second eEPSC induced 50 ms after the first (Beierlein et al, 2003).

Data were evaluated using TIDA 5.24 (HEKA Elektronik, Lambrecht, Germany). sPSCs, mEPSCs and mIPSCs were analyzed using PeakCount V3.2 software (C. Henneberger, Institute of Neurophysiology, Berlin, Germany).

Surgical preparation and in vivo recordings

All experiments were conducted in accordance with the national laws for the use of animals in research and with the European Communities Council Directive 86/609/EEC, and were approved by the local ethical committee (Landesuntersuchungsamt Rheinland-Pfalz 23.177-07/G 10-1-010). Experiments were designed to minimize the number of animals used. Extracellular recordings were performed in the barrel cortex of young adult (P25-32) wild type, and PRG-1$^{+/-}$ mice (for details see (Yang et al, 2009);a four-shank 16-channel electrode (125 µm horizontal shank distance and 50 µm vertical inter-electrode distance, 1-2 MOhm, NeuroNexus Technologies, Ann Arbor, MI, USA) was inserted perpendicularly into the barrel cortex in a depth of 300-400 µm from the cortical surface as described (Yang et al, 2009). To identify the corresponding cortex barrel, single whisker stimulation was performed using a miniature solenoid actuator (modified from (Krupa et al, 2001)). After identifying the corresponding cortex barrel, single whisker stimulation was performed using the described miniature solenoid actuator. This mechanical stimulation produced a 1.2 mm, 16 ms deflection of a single whisker in the rostral-to-caudal direction. In case of paired-pulse stimulation, inter-stimulus interval was set to 500 ms. In each experiment paired stimuli were applied 30 times at 0.05 Hz. For assessment of the ATX inhibition, FP in response to paired-pulse stimulation was measured before and after injection of the ATX-inhibitor HA-130 (personal gift from H. Ovaa). To avoid bias by injection effects, FP in response to paired-

pulse stimulation were measured at the time point when evoked responses were not different to pre-injection values.

Experiments and analysis were performed in a blind fashion. Data were analyzed offline using MATLAB software version 7.7 (MathWorks). For multi-unit activity (MUA) analysis, FP recordings were high-pass filtered (>200 Hz). Seven standard deviations of baseline noise were used as a threshold to detect MUA. MUA burst was defined as a spike train consisting of at least 10 spikes with inter-spike intervals shorter than 50 ms.

Immunocytochemistry

Somatosensory cortex from routine autopsy, C57/Bl6 mice and HEK 293 cells were studied. Colocalization studies were performed using a PRG-1 antibody (custom-made antibody described and characterized by (Trimbuch et al, 2009); dilution: 1:2500) and GAD67 (1:1000; mab5406, Chemicon), ß-Gal (1:2500, ab9361; Abcam), MAP2 (1:1000; mab 3418, Chemicon), Parvalbumin (PV235), Calbindin (D28k) and Calretinin (6B3, all 1:5000; Swant). Confocal images were taken on a Leica SP5. 3D-reconstruction of confocal stacks was performed using Imaris software (Bitplane, Zürich, Switzerland).

Mass Spectrometry analysis of C17 LPA incorporation and degradation

After preparation of HEK293 cells or HEK293 cell lines expressing PRG-1 or PRG-1 mutants, cells were fixed in 2 ml ice cold methanol. For further processing, 1 ml chloroform, 0.5 ml 100 mM HCl were added together with 50 pmol C17:0 S1P (Avanti Polar Lipids, Alabaster, Al, USA), which served as internal control. After vortexing for 5 min, another 1 ml chloroform and 1.3 ml 100mM HCl were added, once again 5 min vortex and then centrifuged at 3,000g for 10 min. The lower phase was transferred to 4 ml screw cap glass vial using a Pasteur pipette and evaporated to dryness under N2 in a fume hood. Sample was resuspended in 100 µl methanol and transferred to autosampler vial with glass insert quantitated by HPLC

electrospray ionization selected ion monitoring mode tandem mass spectrometry as described previously (Salous et al, 2013).

Mass Spectrometry assessment of LPA-species in the CSF. After i.p. inejction PF8380 or its vehicle control as described below, CSF was extracted intraoperatively and LPA analysis was performed with liquid chromatography-electrospray ionization-tandem mass spectrometry (LC-ESI-MS/MS). The LC-MS/MS system consisted in a hybrid triple quadrupole-ion trap QTrap 5500 mass spectrometer (AB Sciex, Germany) equipped with a Turbo-V-source operating in negative ESI mode, an Agilent 1200 binary HPLC pump, column oven (40 °C) and degasser and an HTC Pal autosampler.

Two cycles of lipid-lipid extraction of LPAs were done with 1-buthanol after adding the internal standard LPA17:0 to the sample (50 µl sample, 20 µl internal standard in 500 µl 1-butanol). The combined organic phases were dried under a gentle stream of nitrogen at 45°C and subsequently re-dissolved in 200 µl methanol.

For the chromatographic separation a C18 column (Mercury 20 x 2 mm, 3 µm, 100 Å) and precolumn were used (Phenomenex, Germany). A linear gradient was employed at 400 µl/min. Mobile phase A was water with ammonium formate 50 mM and formic acid (100:0.2, v/v) and mobile phase B acetonitrile/formic acid (100:0.1, v/v), total run time 7 min, injection volume 20 µl. The mass spectrometer operated in the negative ion mode with an electrospray voltage of -4500 V at 350°C. Multiple reaction monitoring (MRM) was used for quantification. The mass transitions were m/z 409→79 (LPA 16:0), m/z 437→153 (LPA 18:0), m/z 431→79 (LPA 18:3) and m/z 457→153 (LPA 20:4) all with a dwell time of 50 ms. All quadrupoles were working at unit resolution. Quantitation was performed with Analyst Software V1.5 (Applied Biosystems) using the internal standard method with

1/concentration2 weighting. The calibration curve was linear over the range of 0.1-500 ng/ml and accuracy was >95%.

Statistical analysis

Since analysis of LPA-species in the cerebospinal fluid (CSF) require profound expertise of the animal neuroanatomy and are very difficult to execute and often biased by vessel puncture and blood contamination during CSF extraction, we excluded values which exceeded the mean +/- 2 SD and were most probably biased by blood contamination (Fig EV4B).

Quantitative phosphorylation assessment

Proteolytic digestion Purified PRG-1 samples were lyophilized down to few microliters and resuspended in 200 µL buffer containing 7 M Urea, 2 M Thiourea, 5 mM DTT, 2% (wt/vol) CHAPS. Subsequently, PRG-1 samples were digested as previously described using modified filter aided sample preparation (Distler et al, 2014; Wisniewski et al, 2009). After proteolytic digest, samples were concentrated, acidified and spiked with heavy peptides (final concentration 25 fmol/µL) prior to LC-MS analysis.

Liquid chromatography-ion mobility separation-mass spectrometry (LC-IMS-MS). Nanoscale UPLC separation was performed using a nanoAcquity UPLC system (Waters Corporation, Manchester, UK) equipped with a nanoAcquity 2G trap column (5 µm, 180 µm x 5 cm, Waters Corporation) and a HSS-T3 C18 analytical column (1.8 µm, 75 µm x 250 mm, Waters Corporation). For analysis, samples (2 µL) were injected onto the trap column. For elution, mobile phase A was water containing 0.1% v/v formic acid and 3% v/v DMSO, while mobile phase B was ACN containing 0.1% v/v formic acid and 3% v/v DMSO. Samples were separated at 45°C with a gradient of 1–60% mobile phase B over 35 min at a flow rate of 300 nL/min. Afterwards, column was rinsed with 90% mobile phase B and re-equilibrated at initial conditions resulting in total analysis times of 60 min.

UPLC system was coupled online to a Synapt G2-S HDMS mass spectrometer (Waters Corporation). Mass spectrometric analysis was performed in positive mode ESI. Samples were analyzed using MS^E as described previously (Distler et al, 2014). Samples were analyzed in three technical replicates.

Data processing. Raw data were analyzed manually using MassLynx V4.1 Software (Waters Corporation). Extracted ion chromatograms of molecular ions were used for quantitative analysis calculating the area under the curve for targeted peptides and their heavy labeled isotopes. Absolute concentrations of target peptides were determined using the spiked in reference peptides.

O-glycosylation assessment

HEK293 cells expressing wtPRG-1 and PRG-1R346T were lysed in 2 x RIPA lysis buffer for 1 h, cleared by centrifugation, and protein concentrations were assessed using an infinite M200 PRO (Tecan, Switzerland) using a Pierce BCA Protein Assay Kit (Thermo Scientific, USA). IP was performed according to standard protocols. Briefly, after pre-clearing an appropriate amount (700 µg protein) of lysate with 50 µl 50% slurry Protein G agarose beads and incubation of 5 µg anti-PRG-1 antibody (Trimbuch et al, 2009) with another 50 µl of 50% slurry Protein G agarose beads, precleared lysate was added to the antibody-bound beads and incubated at 4°C under rotation for 2.5 h. After supernatant removal, beads were washed three times with 2 x RIPA lysis buffer. Finally, each pulldown sample was resuspended in 60 µl lysis buffer and 15 µl 5 x SDS-PAGE loading buffer, heat shocked in a thermomixer (Eppendorf, Germany) at 95°C for 10 min and analyzed by western blotting according to standard procedures. Briefly, samples were separated by SDS-PAGE using 10% polyacrylamide gels, and blotted on a nitrocellulose membrane (0.45 µm; BIO-RAD, USA) by semi-dry transfer cell (BIO-RAD, USA) at 100 mA for 40 min. Blots were blocked overnight at 4°C in 10% non-fat dry milk, washed twice, and incubated at room temperature

(RT) for 3 h with a mouse anti-O-GlcNAc antibody (1:1000 dilution; CTD110.6, Cell Signaling, USA) in 1% non-fat BSA. After three washes, membranes were incubated with a donkey anti-mouse IgG horseradish peroxidase-conjugated antibody (1:5000 dilution; dianova, Germany) in 1% non-fat BSA at RT for 1 h. Immunoreactive bands were detected using SuperSignal West Femto Maximum Sensitivity Substrate kit (Thermo Scientific, USA) according to standard protocols. To test for the amount of blotted PRG-1 protein, membranes were mildly stripped (stripping buffer: 15 g glycine, 1 g SDS, 10 ml Tween 20, adjusted to pH to 2.2), blocked in 10% non-fat dry milk in at 4°C overnight, washed twice, and then incubated 3 h at room temperature (RT) with a rabbit anti-PRG-1 antibody (1:1000 dilution; Pineda, Germany) in 1% non-fat BSA. After washing, membranes were incubated with a donkey anti-rabbit IgG horseradish peroxidase-conjugated antibody (1:5000 dilution; dianova, Germany) in 1% non-fat BSA at RT for 1h. Immunoreactive bands were detected using enhanced chemiluminescence (ECL) western blotting detection reagents (GE Healthcare, UK).

TF-LPA uptake experiments

Plasmids Construction and Cell Lines Establishment

PRG-1 cDNA was amplified by PCR and cloned in pNTAP-B (Stratagene). The PRG-1 R346 site-directed mutagenesis was achieved by overlap extension using QuickChangeII XL Site-Directed mutagenesis kit (Stratagene), confirmed by sequencing and subcloned into pCAG-IRES-GFP (Addgene); PRG1 cDNA was also cloned into the same site as the mutant counterpart. HEK293 cell lines expressing different versions of PRG1 ($PRG1^{wt}$, $PRG1^{R346T}$) were established by continual G418 selection (600µg/ml). Primer sequences are available on request. For immunofluorescence visualization cells were fixed in 4% PFA and stained with DAPI (1:10.000 Invitrogen, Darmstadt, Germany). Pictures were acquired on a Olympus BX51 fluorescence microscope using a 60x oil objective (NA 1,4).

Preparation and transfection of primary neuronal cultures

Primary neuronal cultures were prepared and transfected as described (Brandt et al, 2007). Briefly, pups derived from PRG-$1^{+/-}$ termed mated pregnant mice were prepared in separate tubes allowing preparation and cultivation of wild-type, PRG-$1^{+/-}$ and PRG-$1^{-/-}$ neurons from the same litter. After 5 DIV (days in vitro), genotyped PRG-$1^{-/-}$ neurons were transfected with a construct (pCAG) coding either for wild-type PRG-1 (PRG-1^{WT}) or for the mouse homologue (PRG-1^{R346T}) of the described “human” mutation R345T. Transfection was performed using Effectene (Qiagen) according to the manufacturer`s instruction. The construct contained an EGFP-IRES cassette which served to identify the transfected neurons among the surrounding PRG-$1^{-/-}$ neurons. For control experiments, wild type or PRG-$1^{-/-}$ neurons were transfected with a construct expressing only the EGFP-IRES cassette. To allow for expression and protein production, experiments were performed 2 days after transfection. Functional analyses of wild-type and PRG-$1^{+/-}$ neurons were performed using the same cell culture procedure at DIV 10.

Optical detection of neuronal TF-LPA uptake

TF-LPA (Avanti Polar Lipids, Alabaster, Al, USA; (Saunders et al, 2011)) uptake in primary neurons was quantified using an inverse fluorescence microscope (IX81, Olympus, Japan), equipped with a high-speed camera (XM10, Olympus). Images were acquired with a high numerical aperture 20 x objective (NA 0,7; UCPLFLN, Olympus, Japan), and a GFP filter coupled to a light source (USHIO, Olympus, Japan). High resolution bright field and fluorescent images were acquired before and after the time lapse imaging.

Time lapses were acquired at a resolution of 688*515 pixels at a frequency of 20 Hz, for 300 s. At about 10 s upon the commencement of the time lapse, 1 ml of 10µM TF-LPA (Avanti Polar Lipids, USA) was added to the respective well. TF-LPA uptake could be measured 10 s upon TF-LPA stimulation.

Image data was analyzed in a blind fashion and was performed by defining regions of interests (ROIs) within the neurons on the bright-field image acquired before the time lapse, using ImageJ (NIH, USA). ROIs were copied to the fluorescence images, and intensity values of all pixels within a given ROI were averaged. The time courses of the averaged intensity values of all ROIS were imported to Igor software (WaveMetrics, Portland, USA) for further analysis. All traces represent relative changes in fluorescence ($\Delta f/f$) in a given ROI. No smoothing or filtering of the data was conducted. Changes of fluorescence intensity upon administration of TF-LPA were defined as differences from the averaged baseline prior to TF-LPA application, to the new steady state 10 s after TF-LPA application.

Statistical analysis was conducted using SPSS (USA) or GraphPad Prism (USA) software. First, data sets from all conditions, comprising of individual ROIs each integrating intensity values of one neuronal soma, were tested for normal distribution using the parameter free one-sample Kolmogorov-Smirnov test (K.-S.-test). In cases where normal distribution of data could be assumed ($p > 0.05$), the parametric two-tailed Student's t-test was employed to compare means. Transfection experiments where non-transfected (PRG-$1^{-/-}$) and transfected (either PRG-1^{wt} or PRG-1^{R346T}) neurons were analyzed using an ANOVA test for repeated measurements and a Bonferroni post-hoc test to compare the means.

Behavioral analyses

All experiments were conducted in accordance with the national laws for the use of animals in research and with the European Communities Council Directive 86/609/EEC, and were approved by the local ethical committee (Landesuntersuchungsamt, Rheinland Pfalz, Germany, #23 177-07/G 12-1-096). Experiments were designed to minimize the number of animals used. PRG-$1^{+/-}$ males (het, n=14) and wild type (wt, n=12) littermates in the 10th generation backcrossed to C57Bl/6 were housed at 3–5 per cage in a room with a 12-h light-dark cycle (lights on at 06:00 h) with ad libitum access to food and water. Mice were 12-14

weeks of age. Inter-assay interval was 1-2 days. Behavioral tests were conducted in a blind fashion during the light phase of the day from 09:00 until 17:00 h.

Social interaction index

The behavior of the wildtype and PRG-$1^{+/-}$ mice was assessed in a three chambered box as described (Radyushkin et al, 2009). The social interaction index was calculated using the following formula: $((Time_{stranger} / Time_{stranger} + Time_{empty})\ x100)\ -50$.

Tail suspension test (TST)

Behavioral despair was measured by suspending the mouse by the tail (attached to the bar by adhesive tape) on a horizontal aluminum bar attached to the top of a box-like enclosure (33×33×32 cm). The distance between the tip of the nose of the mouse and the floor was approximately 20 cm. Video fragments were recorded and the time spent immobile during the 6 min was analyzed by the video tracking system “EthoVision XT 8”.

Restraint stress

Two weeks after finishing of behavioral tests at normal conditions restraint stress was applied. Animals were restrained for 2 h in a modified 50 ml, clear polypropylene tube (diameter 3 cm, 12 cm long) with multiple air holes for ventilation. 15 minutes after the restraint stress mice were retested in the tail suspension test.

Pre-pulse Inhibition

Mice: Separate cohort of male and female mice of 5 months old was used for this experiment. Mice were single-housed in standard mouse cages with 12/12h light-dark cycle and *ad libitum* access to food and water. 10-19 animals were used per one experimental group.

PPI: Test was performed as described earlier (Radyushkin et al, 2009). Briefly, mice were placed in small metal cages (90 × 40 × 40 mm) to restrict major movements. The cages were equipped with a movable platform floor attached to a sensor that records vertical movements of the floor. The cages were placed in four sound-attenuating isolation cabinets (Med Associates, St.Albans, VT, USA). Startle reflexes were evoked by acoustic stimuli delivered from a loudspeaker that was suspended above the cage and connected to an acoustic generator. The startle reaction to an acoustic stimulus, which evokes a movement of the platform and a transient force resulting from this movement of the platform, was recorded with a computer during a recording window of 380 milliseconds (beginning with the onset of pre-pulse) and stored for further evaluation. An experimental session consisted of a 2-min habituation to 65 dB background white noise (continuous throughout the session), followed by a baseline recording for 1 min. After baseline recording, 8 pulse-alone trials using startle stimuli of 120 dB intensity and 40 milliseconds duration were applied in order to decrease the influence of within-session habituation. These data were not included in the analysis of the pre-pulse inhibition. For tests of pre-pulse inhibition, the 120 dB/40 milliseconds startle pulse was applied either alone or preceded by a pre-pulse stimulus of 70, 75 or 80 dB intensity and 20 milliseconds duration. An interval of 100 milliseconds with background white noise was employed between each pre-pulse and pulse stimulus. 10 trials of each kind (in total 40 trials) were applied in a pseudorandom order with variable inter-trial interval ranging from 8 to 22 seconds. Amplitudes of the startle response were averaged for each individual animal, separately for each type of trials (i.e. stimulus alone or stimulus preceded by a pre-pulse f 3 intensity). Pre-pulse inhibition was calculated as the percentage of the startle response using the following formula: PPI (%) = 100 – [(startle amplitude after pre-pulse and pulse / startle amplitude after pulse only) × 100].

Pharmacological treatment

Animals were treated using the in-vivo potent autotaxin inhibitor PF 8380 (Cayman Chemical Company, Ann Arbor, MI, USA). On the basis of the data presented by (Gierse et al, 2010), and our own pilot experiments we selected the dose of 30 mg/KG body weight for treatment. The drug was dissolved in dimethylsulfoxid (DMSO) as a carrier and animals received a single i.p. injection 3 hours prior to PPI. The volume of the injection was 25 µl. Control animals were injected with the same volume of DMSO.

Behavioral data were analyzed by 2-Way ANOVA for repeated measures using GraphPad Prism 6 software (GraphPad Software, Inc., CA, USA). A p value below 0.05 was considered to be significant (* P< 0.05; **P< 0.01).

Appendix References

Agmon A, Connors BW (1991) Thalamocortical responses of mouse somatosensory (barrel) cortex in vitro. *Neuroscience* **41:** 365-379

Beierlein M, Gibson JR, Connors BW (2003) Two dynamically distinct inhibitory networks in layer 4 of the neocortex. *Journal of neurophysiology* **90:** 2987-3000

Brandt N, Franke K, Rasin MR, Baumgart J, Vogt J, Khrulev S, Hassel B, Pohl EE, Sestan N, Nitsch R, Schumacher S (2007) The neural EGF family member CALEB/NGC mediates dendritic tree and spine complexity. *The EMBO journal* **26:** 2371-2386

Distler U, Kuharev J, Navarro P, Levin Y, Schild H, Tenzer S (2014) Drift time-specific collision energies enable deep-coverage data-independent acquisition proteomics. *Nature methods* **11:** 167-170

Gierse J, Thorarensen A, Beltey K, Bradshaw-Pierce E, Cortes-Burgos L, Hall T, Johnston A, Murphy M, Nemirovskiy O, Ogawa S, Pegg L, Pelc M, Prinsen M, Schnute M, Wendling J, Wene S, Weinberg R, Wittwer A, Zweifel B, Masferrer J (2010) A novel autotaxin inhibitor reduces lysophosphatidic acid levels in plasma and the site of inflammation. *The Journal of pharmacology and experimental therapeutics* **334:** 310-317

Krupa DJ, Brisben AJ, Nicolelis MA (2001) A multi-channel whisker stimulator for producing spatiotemporally complex tactile stimuli. *Journal of neuroscience methods* **104:** 199-208

Radyushkin K, Hammerschmidt K, Boretius S, Varoqueaux F, El-Kordi A, Ronnenberg A, Winter D, Frahm J, Fischer J, Brose N, Ehrenreich H (2009) Neuroligin-3-deficient mice:

model of a monogenic heritable form of autism with an olfactory deficit. *Genes, brain, and behavior* **8:** 416-425

Salous AK, Panchatcharam M, Sunkara M, Mueller P, Dong A, Wang Y, Graf GA, Smyth SS, Morris AJ (2013) Mechanism of rapid elimination of lysophosphatidic acid and related lipids from the circulation of mice. *J Lipid Res* **54:** 2775-2784

Saunders LP, Cao W, Chang WC, Albright RA, Braddock DT, De La Cruz EM (2011) Kinetic analysis of autotaxin reveals substrate-specific catalytic pathways and a mechanism for lysophosphatidic acid distribution. *The Journal of biological chemistry* **286:** 30130-30141

Trimbuch T, Beed P, Vogt J, Schuchmann S, Maier N, Kintscher M, Breustedt J, Schuelke M, Streu N, Kieselmann O, Brunk I, Laube G, Strauss U, Battefeld A, Wende H, Birchmeier C, Wiese S, Sendtner M, Kawabe H, Kishimoto-Suga M, Brose N, Baumgart J, Geist B, Aoki J, Savaskan NE, Brauer AU, Chun J, Ninnemann O, Schmitz D, Nitsch R (2009) Synaptic PRG-1 modulates excitatory transmission via lipid phosphate-mediated signaling. *Cell* **138:** 1222-1235

Wisniewski JR, Zougman A, Nagaraj N, Mann M (2009) Universal sample preparation method for proteome analysis. *Nature methods* **6:** 359-362

Yang JW, Hanganu-Opatz IL, Sun JJ, Luhmann HJ (2009) Three patterns of oscillatory activity differentially synchronize developing neocortical networks in vivo. *The Journal of neuroscience : the official journal of the Society for Neuroscience* **29:** 9011-9025
