# Supplementary material for: Molecular cause and functional impact of altered synaptic lipid signaling due to a prg‐1 gene SNP
Source: EMBO Mol Med. 2015 Dec 15;8(1):25–38. doi: 10.15252/emmm.201505677 (PMC4718157; doi:10.15252/emmm.201505677)
Supplement: Supplementary file 2 — Expanded View Figures PDF [file EMMM-8-25-s002.pdf]

Expanded View Figures

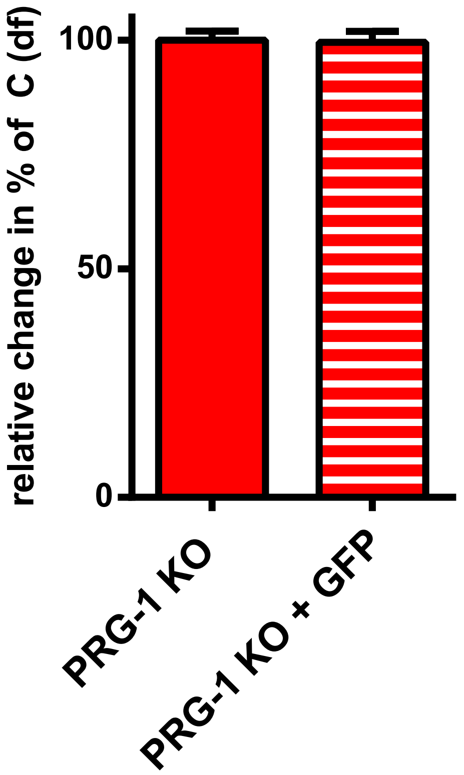

Figure EV1. GFP expression in PRG-1$^{-/-}$ neurons does not influence TF-LPA uptake.

Quantitative assessment of non-transfected PRG-1$^{-/-}$ and GFP-transfected PRG-1$^{-/-}$ neurons revealed no significant difference in TF-LPA uptake (n = 110 non-transfected PRG-1$^{-/-}$ and 34 GFP-transfected PRG-1$^{-/-}$ neurons, unpaired t-test).

Data information: Bar diagrams represent mean ± SD.

© 2015 The Authors

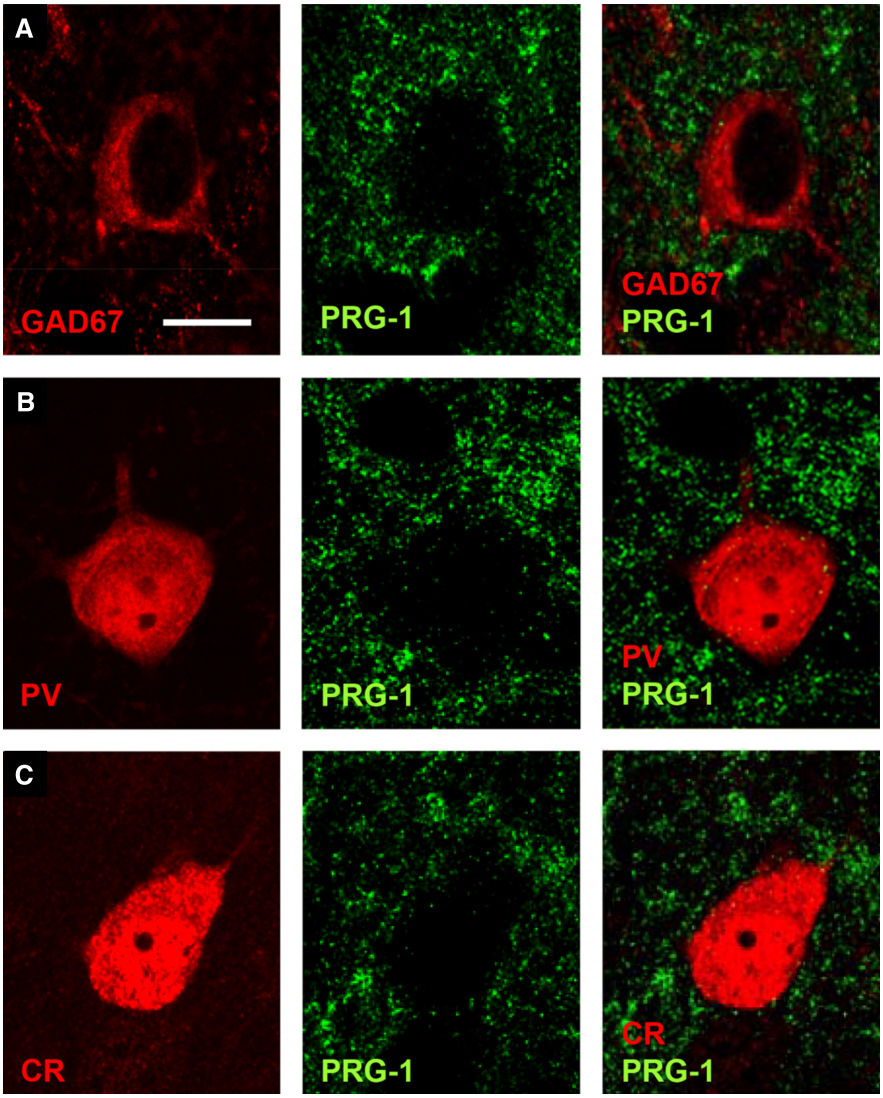

Figure EV2. PRG-1 is not expressed in GABAergic neurons in the somatosensory cortex.

A–C PRG-1 expression was not detected in inhibitory GAD67 (A)-, parvalbumin (PV, B)-, or calretinin (CR, C)-positive interneurons of the mouse somatosensory barrel field cortex (S1BF). Scale bar: 10 μm.

 © 2015 The Authors

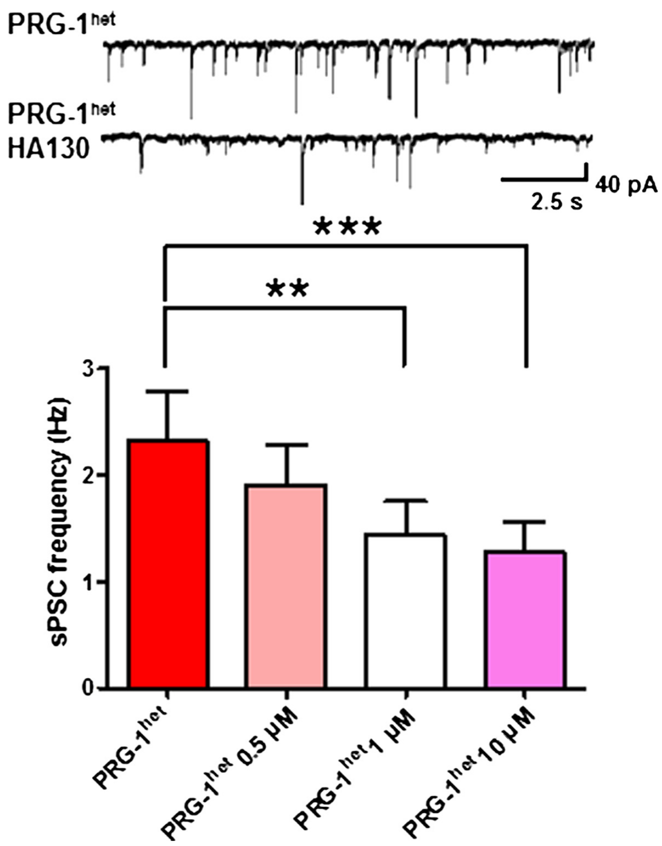

Figure EV3. ATX inhibition decreases neuronal excitation in single cells.
ATX inhibition in PRG-1$^{+/-}$ mice, which display a neuronal hyperexcitability, revealed a significant decrease of spontaneous postsynaptic currents (sPSC) at increasing concentrations of the ATX blocker HA130. sPSCs are the sum of the overall excitatory and inhibitory input at the single neuron level and reflect the excitation/inhibition (E/I) balance (n = 7, repeated-measures ANOVA (P = 0.0002) with Bonferroni's multiple comparison test; $^{**}P < 0.01$, $^{***}P < 0.001$). Bar diagrams represent mean ± SEM.

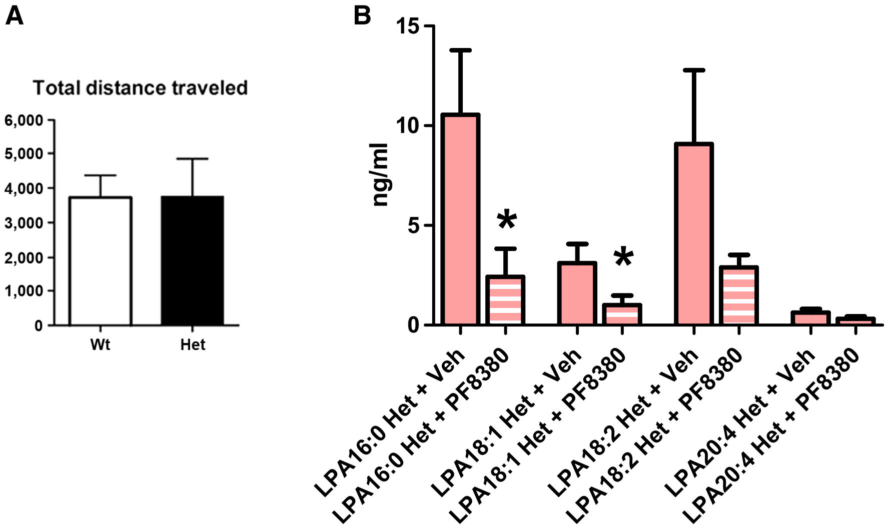

Figure EV4. Decreased LPA levels in the CSF after ATX inhibition.

- A To exclude bias in the social alteration by lower motility, total distance traveled by the mice of the different genotypes was analyzed and was not different between genotypes (Mann–Whitney U-test; n = 13 wt and 15 PRG-1 het mice).
- B LPA levels in the cerebrospinal fluid measured by mass spectrometry 3 h after i.p. application of vehicle (DMSO) or of the *in vivo* ATX inhibitor PF8380 in PRG-1$^{+/-}$ mice revealed a significant decrease of the main LPA subtypes (n = 6 samples pooled from 2 animals per sample and n = 5 for LPA18:2 Het + PF8380; unpaired, one-tailed t-test, for LPA16:0 $^{*}P$ = 0.0219, LPA 18:1 $^{*}P$ = 0.0398; due to sophisticated CSF extraction, which may be biased by blood contamination, values exceeding mean + 2 × SD were excluded).

Data information: Bar diagrams represent mean ± SEM.

© 2015 The Authors

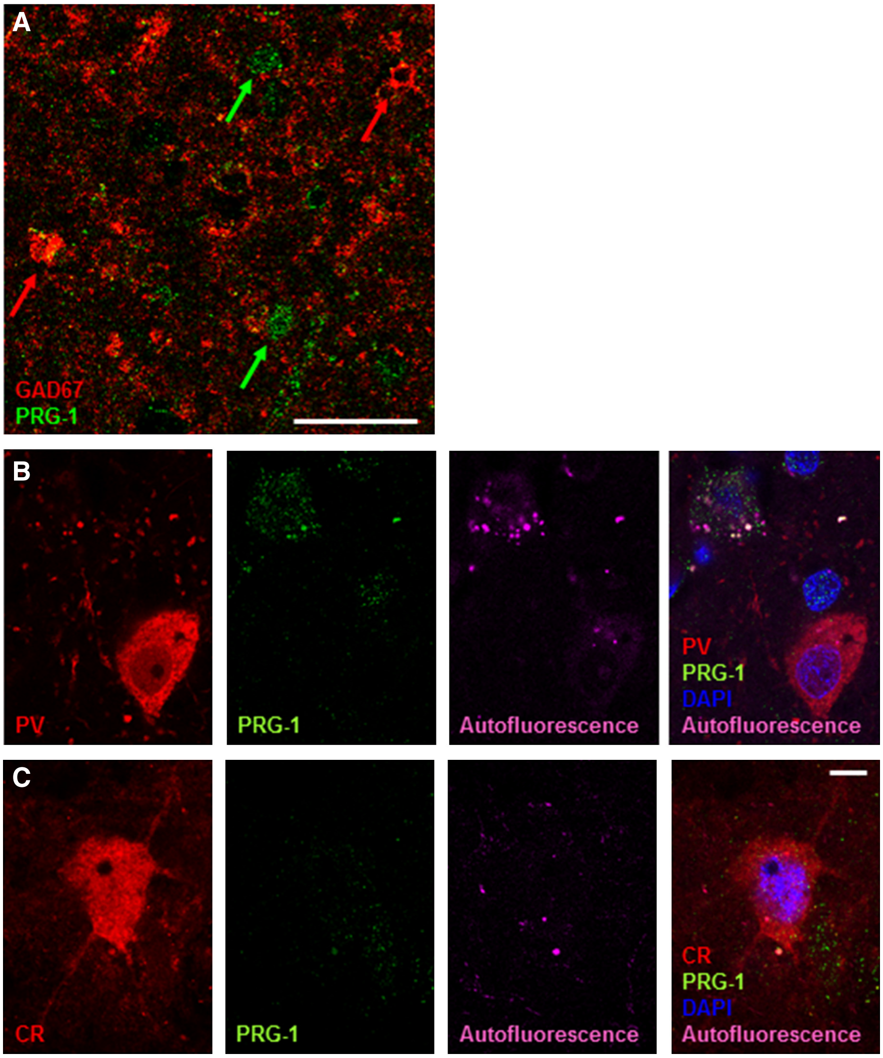

Figure EV5. PRG-1 is not expressed in cortical interneurons of the human somatosensory cortex.

A Overview of PRG-1 and GAD67 expression shows distinct non-overlapping localization. Remark the clear non-overlapping delineation of PRG-1-expressing neurons (green arrows) and GAD67-interneurons (red arrows).

B, C Higher magnification shows a parvalbumin (PV)- and a calretinin (CR)-positive interneuron confirming no expression of PRG-1 in these interneurons. Since autofluorescence is a common feature in human tissue and might lead to interpretation bias, we have illustrated the extent of autofluorescence in each depicted neuron by using a 633-nm laser line which excites only autofluorescent material but not the secondary antibodies labeled with Alexa 488 (against PRG-1 antibody) and 568 (against PV and CR antibodies) which were excited with a laser line of 488 nm and 568 nm, respectively. Note the PRG-1 expression in the nearby located PV-negative neuron in (B).

Data information: Scale bars: 50 μm (A), 5 μm (B, C).

 © 2015 The Authors
